# Trends of and factors associated with access to residential toilets among the middle-aged and elderly in rural China from 2011 to 2018

**DOI:** 10.1186/s12889-022-12739-3

**Published:** 2022-03-02

**Authors:** Qun Wang, Huiyuan Cao, Shuo Zhang

**Affiliations:** 1 Faculty of Humanities and Social Sciences, Dalian University of Technology, Dalian, China; 2 School of Public Administration, College of Management and Economics, Tianjin University, Tianjin, China; 3 Institute of Complex Systems On Decision and Control, Dalian University of Technology, Dalian, China

**Keywords:** Access to toilet, Trend, The elderly, Rural areas

## Abstract

**Background:**

At the global and country levels, several important sanitation improvement initiatives were launched in the last decade. This study aimed to explore the temporal trend of and factors associated with access to residential toilets among the middle-aged and elderly in rural China from 2011 to 2018.

**Methods:**

This study used the 2011, 2013, 2015, and 2018 data of China Health and Retirement Longitudinal Study (CHARLS). CHARLS was conducted among adults aged ≥ 45 years in 28 provinces of China. We used descriptive statistics and logistic regressions for data analysis.

**Results:**

We found that residential toilet coverage increased by about 6% among population aged ≥ 45 years in rural China from 2011 to 2018. The coverage of flushable toilets and toilets with seats among this sector of the population increased by more than 10% during this period. We also found that being female, higher levels of education, higher annual per capita household consumption, having running water in the residence, larger household size, and better health status were significantly associated with an increased likelihood of residential toilet ownership among population aged ≥ 45 years in rural China.

**Conclusions:**

China made progress in sanitation improvement in rural areas from 2011 to 2018. However, considering the current coverage levels of residential toilets and the vulnerable subgroups who are more prone to toilet deprivation in rural areas, we suggest to the government to implement further targeted toilet improvement interventions to ensure universal coverage of sanitation facilities for the whole of the Chinese population.

**Supplementary Information:**

The online version contains supplementary material available at 10.1186/s12889-022-12739-3.

## Background

Sanitation is of vital importance for health maintenance. However, approximately 2 billion people globally still lack basic sanitation facilities. Of these 2 billion, 70% live in rural areas [1]. Deprivation of proper sanitation facilities may result in the spread of many infectious diseases. The world has missed the Millennium Development Goal target of halving the proportion of people without sustainable access to sanitation by 2015. In 2015, the United Nations General Assembly set up the Sustainable Development Goals (SDGs). One of these goals was to ensure access to sanitation for all by 2030. Most low- and middle-income countries (LMICs) are taking special efforts [1, 2] to achieve this specific SDG target.

As one of the largest LMICs, China has long been devoted to sanitation improvement. Since the 1950s, the Chinese government, aiming to promote environmental health in rural areas, has paid great attention to toilet provision, regarding it as one of the most important sanitation facilities [3]. From 1978 to 2002, China set the goal related with the toilet coverage in rural areas and included toilet improvement in the national economic and social development plans. From 2003 to 2012, the government further promoted toilet reforms in rural areas and made toilet improvement one of the important targets of rural reform and development. From 2009 to 2011, China implemented a new round of healthcare reforms and promoted toilet improvement in rural areas as a major public health service project. Since 2013, the country has entered a new era of comprehensively implementing and deepening toilet reforms in rural areas. The National Environmental Sanitation Action Plan (2015–2020) set the following goals for sanitary toilet coverage: 75% by 2015, 85% by 2020, and 100% by 2030. In 2015, President Xi Jinping explicitly proposed that a “toilet revolution” should take place in rural China in order to provide the entire rural population with access to sanitary toilets [4]. In the last two decades, China has made progress on toilet provision. According to the estimates from National Health Commission of the People’s Republic of China, between 2000 to 2017 the coverage of sanitary toilets in rural China increased from 40.3% to 81.8% [5].

In parallel with global and country-level sanitation initiatives, access to sanitation has generated heated discussion in the academic community. Systematic studies on access to sanitation are of key importance for policy makers to design effective interventions for a targeted population. Globally, most studies on sanitation were based in LMICs. A large number of these studies focused on the effect of access to sanitation on health inequities [6–8], or on the occurrence and disease burden of some infectious diseases [9–11], or on other social indicators of development (e.g. student enrollment, educational efficiency, and violence against women) [12–14]. Another large number of these studies were centered on factors associated with or inequality in access to sanitation, with most of them using cross-sectional data. And these studies showed that socially disadvantaged groups (those with lower income and education, etc.) were more likely to suffer from no or poor access to sanitation [15–24]. A small number of such studies relied on longitudinal data with special focus on trends of access to sanitation [25–29]. However, to our best knowledge, no studies have been conducted that systematically explore the changes of and factors associated with access to sanitation in rural China after 2013, especially after the implementation of the “toilet revolution” campaign. The existing studies using longitudinal data based in China both focused on the trends of and factors associated with access to toilet facilities before 2012 [26, 27, 29]. In addition, all the studies on access to toilet facilities based in China looked at the general population without paying special attention to the middle-aged and elderly population, a very large group of the population in a country with a severe aging crisis. This paper aims to fill the gap in this field by using the nationally representative China Health and Retirement Longitudinal Study (CHARLS) data from 2011 to 2018.

## Methods

### Source of data

We used data from the 2011, 2013, 2015, and 2018 waves of CHARLS, which is designed to be comparable with both the Health and Retirement Study in the USA and related aging surveys around the world. As a national longitudinal household survey, CHARLS was conducted among adults aged ≥ 45 years in 28 provinces, 150 countries/districts, and 450 villages/urban communities across China. Among the selected 450 villages/urban communities, 52.67% were in rural areas and 47.33% in urban areas. CHALRS adopted a multi-stage stratified probability proportionate to size sampling. The main information collected by CHARLS included demographic background, household and family information, health status and function, health care and insurance, work, retirement and pension, income, expenditure and assets, biomarkers and so on [30].

In this study, we used six sections of data in CHARLS, including demographic background, health status and function, health care and insurance, household roster, household income, and housing characteristics. After merging data in all these sections, we kept the data that had records in all these six sections, i.e., 17,403, 18,375, 20,860, and 19,732 in the 2011, 2013, 2015, and 2018 rounds, respectively. We then kept the observations with rural Hukou and aged ≥ 45 years. Finally, a total of 13,240 in 2011 (76.08% of the whole sample in the year), 13,856 in 2013 (75.41% of the whole sample in the year), 12,909 in 2015 (61.88% of the whole sample in the year), and 11,316 observations in 2018 (57.35% of the whole sample in the year) were included in the final analysis.

### Variables and their measurement

This study had three outcome variables and were all expressed in dummy variables. The first outcome variable was defined as whether the individuals surveyed had a residential toilet. The second outcome variable was defined as whether the toilet was flushable. The third outcome variable was defined as whether the toilet had a seat.

In addition, this study included sociodemographic information, family characteristics and health conditions variables to stratify different population subgroups. Sociodemographic information included gender, age, education level, marital status, region, and whether the residence had running water. Family characteristics included household size and annual per capita household consumption. Health conditions included self-reported health and activities of daily living (ADLs). Most of the variables were self-explanatory except annual per capita household consumption, ADLs, and region.

The variable of annual per capita household consumption was calculated based on a set of related items. CHARLS collected data on household food consumption over the past seven days, as well as data on household nonfood consumption over the last month and the last year. Household food consumption included purchased food and food eaten from own production, meals eaten out, alcohol, and tobacco. Household nonfood consumption over the last month included communication, utilities, fuels, payment for servants, local transportation, daily necessities, and entertainment. Household nonfood consumption over the last year included clothing, bedding, long-distance travel, heating, durable goods, education and training, medical expenses, fitness, beauty, vehicle purchase, maintenance and repair, taxes and fees, automobiles, electronics, property management fees, and donations. We converted the household food consumption over the past seven days and the household consumption over the last month into annual costs to enable the calculation of the total annual household consumption. All expenditure was expressed in Chinese Yuan (CNY). In order to account for expenditure differences due to household size, per capita expenditure was calculated by dividing aggregated household expenditure by household size.

In line with previous studies [31], ADLs were measured by six representative daily activities: dressing, bathing, eating, getting into or out of bed, using the toilet, and controlling urination and defecation. Each daily activity question had four answer categories: able to perform independently without difficulty, able to perform independently with some difficulty, need some help to perform, and completely unable to perform. If the respondent chose the first two answer categories of one activity, then he/she was defined as “independently” performing the activity. If the respondent chose the last two answer categories of one activity, then he/she was defined as “dependently” performing the activity. All respondents were classified into two groups: no ADL disability (the status without any dependent activities) and with ADL disability (the status with at least one dependent activity).

In line with the four major economic regions defined by National Bureau of Statistics, region was referred to as whether the respondent resided in Northeastern, Western, Central, or Eastern China. Northeastern region includes Heilongjiang, Jilin, and Liaoning. Western region includes Xinjiang, Gansu, Sichuan, Chongqing, Shaanxi, Guizhou, Yunnan, Guangxi, Qinghai, and Inner Mongolia. Central region includes Shanxi, Anhui, Jiangxi, Henan, Hunan, and Hubei. Eastern region includes Beijing, Tianjin, Hebei, Shanghai, Jiangsu, Zhejiang, Fujian, Shandong, and Guangdong [32].

### Analytical approach

We first applied descriptive statistics to report the temporal trend of residential toilet ownership and the coverage of flushable toilets and seated toilets among rural respondents aged ≥ 45 years in China from 2011 to 2018. A Chi- square test was used to initially analyze the factors related to residential toilet ownership.

Similar with previous studies [28], we then used logistic regression on data in each year to further analyze the factors associated with residential toilet ownership. We also used logistic regression to explore the factors influencing access to toilets with seats and flushable toilets using CHARLS 2018 data. We used the command of “collin” to check multicollinearity in the logistic models. All statistical significance decisions were based on 2-tailed P values and the significance level was chosen at 0.05. We used Stata to conduct all statistical analyses.

## Results

Table 1 and 2 show the characteristics of the complete sample and the sample without residential toilets. Of the whole sample, over 50% were women. The average age of the entire sample was 59.20, 60.22, 61.23, and 63.90 in 2011, 2013, 2015, and 2018, respectively. Overall, the level of education among the whole sample was low, with more than 85% having a middle school education or below. As for marital status, the vast majority were married. As for water, the percentage of respondents having residential running water increased from 55% in 2011 to 77% in 2018. On average, the household size of the whole sample was 2.28, 2.28, 2.09, and 2.10 in 2011, 2013, 2015, and 2018, respectively. The median of annual per capita household consumption was 7,818.80 CNY in 2011, 10,303.65 CNY in 2013, 11,548.81 CNY in 2015, and 11,576.10 CNY in 2018. Among the respondents, more than 90% had no ADL disability and around 70% reported fair or good health status. The sample in each selected province in each year is shown in Appendix Table 1.

**Table 1:** Descriptive sample characteristics I

	2011					2013				
	Entire sample		No toilet sample		*P* value	Entire sample		No toilet sample		*P* value
	N(1)	%	N(2)	(2)/(1)		N(3)	%	N(4)	(4)/(3)	
**Gender**					0.311					0.344
Male	6326	47.78	1891	29.89		6482	46.78	1714	26.44	
Female	6903	52.14	2008	29.09		7352	53.06	1912	26.01	
Missing	11	0.08				22	0.16	3	13.64	
**Age**					< 0.001					0.003
45–59	8003	60.45	2258	28.21		6720	48.50	1685	46.43	
60–69	3113	23.51	933	29.97		4029	29.08	1066	26.46	
70 and above	2124	16.04	708	33.33		3107	22.42	878	28.26	
**Education Level**					< 0.001					0.004
Illiterate	4318	32.61	1421	32.91		4159	30.02	1153	27.72	
Primary or middle school	8100	61.18	2253	27.81		8024	57.91	2057	25.64	
High school or above	806	6.09	218	27.05		790	5.70	180	22.78	
Missing	16	0.12	7	43.75		883	6.37	239	27.07	
**Marital status**					< 0.001					< 0.001
Married	11,480	86.71	3308	28.82		12,002	86.62	3078	25.65	
Divorced/widowed/never married	1760	13.29	591	33.58		1854	13.38	551	29.72	
**Region**					< 0.001					< 0.001
Northeastern	659	4.98	490	74.36		730	5.27	448	61.37	
Western	4458	33.67	1193	26.76		4669	33.70	1210	25.92	
Central	3813	28.80	1026	26.91		3960	28.58	958	24.19	
Eastern	4610	32.55	1190	27.61		4497	32.46	1013	22.53	
**Having running water**					< 0.001					< 0.001
No	5896	44.53	2369	40.18		4736	34.18	1683	35.54	
Yes	7326	55.33	1526	20.83		9106	65.72	1944	21.35	
Missing	18	0.13	4	22.22		14	0.10	2	14.29	
**Household size**					< 0.001					< 0.001
Living alone	666	5.03	274	41.14		639	4.61	249	38.97	
2–4 people	8226	62.13	2508	30.49		8639	62.35	2451	28.37	
5 people and above	4348	32.84	1117	25.69		4578	17.76	929	20.29	
**SES**					< 0.001					0.008
Lowest 25%	3313	25.02	1091	32.93		3465	25.01	967	27.91	
Lower 25%	3307	24.98	1000	30.24		3463	24.99	922	26.62	
Higher 25%	3311	25.01	991	29.93		3464	25.00	896	25.87	
Highest 25%	3309	24.99	817	24.69		3464	25.00	844	24.36	
**ADL**					0.003					< 0.001
No ADL disability	12,446	94.00	3628	29.10		13,006	93.87	3363	25.86	
With ADL disability	794	6.00	271	34.13		850	6.13	266	31.29	
**Self-reported health**					< 0.001					< 0.001
Good	2928	22.11	812	27.73		2972	21.45	735	24.73	
Fair	6308	47.64	1720	27.27		6742	48.66	1642	24.35	
Poor	3986	30.11	1363	34.19		3545	25.58	1086	30.63	
Missing	18	0.14	4	22.22		597	4.31	166	27.81

**Table 2:** Descriptive sample characteristics II

	2015					2018				
	Entire sample		No toilet sample		*P* value	Entire sample		No toilet sample		*P* Value
	N(7)	%	N(8)	(8)/(7)		N(5)	%	N(6)	(6)/(5)	
**Gender**					0.639					0.396
Male	6015	46.60	1312	21.81		5191	46.60	1243	23.95	
Female	6893	53.40	1480	21.47		6125	53.40	1425	23.27	
Missing	1	0.01								
**Age**					< 0.001					< 0.001
45–59	5652	43.78	1129	40.44		4030	35.61	867	21.51	
60–69	4258	32.98	918	21.56		4336	38.32	1060	24.45	
70 and above	2999	23.23	745	24.84		2950	26.07	741	25.12	
**Education Level**					0.107					< 0.001
Illiterate	3863	29.92	853	22.08		3372	29.80	880	26.10	
Primary or middle school	7627	59.08	1644	21.56		7267	64.22	1658	22.82	
High school or above	727	5.63	135	18.57		677	5.98	130	19.20	
Missing	692	5.36	160	23.12						
**Marital status**					< 0.001					0.037
Married	11,039	85.51	2290	20.74		9429	83.32	2188	23.21	
Divorced/widowed/never married	1869	14.48	502	26.86		1887	16.68	480	25.44	
Missing	1	0.001								
**Region**					< 0.001					< 0.001
Northeastern	666	5.16	404	60.66		590	5.21	372	63.05	
Western	4322	33.48	924	21.38		3779	33.40	986	26.09	
Central	3697	28.64	694	18.77		3267	28.87	650	19.90	
Eastern	4224	32.72	770	18.23		3680	32.52	660	17.93	
**Having running water**					< 0.001					< 0.001
No	3675	28.47	1108	30.15		2626	23.21	997	37.97	
Yes	9232	71.52	1684	18.24		8688	76.78	1671	19.23	
Ming	2	0.02	0	0		2	0.02	0	0	
**Household size**					< 0.001					< 0.001
Living alone	679	5.26	229	33.73		541	4.78	178	32.90	
2–4 people	10,401	80.57	2285	21.97		9149	80.85	2171	23.73	
5 people and above	1829	14.17	278	15.20		1626	14.37	319	19.62	
**SES**					< 0.001					< 0.001
Lowest 25%	3229	25.01	795	24.62		2832	25.03	803	28.35	
Lower 25%	3226	24.99	739	22.91		2830	25.01	698	24.66	
Higher 25%	3227	25.00	642	19.89		2825	24.96	622	22.02	
Highest 25%	3227	25.00	616	19.09		2829	25.00	545	19.26	
**ADL**					< 0.001					0.002
No ADL disability	11,948	92.56	2534	21.21		10,378	91.71	2408	23.20	
With ADL disability	961	7.44	258	26.85		938	8.29	260	27.72	
**Self-reported health**					< 0.001					< 0.001
Good	2748	21.44	548	19.94		2353	20.8	532	22.61	
Fair	6343	49.14	1269	20.01		5175	45.73	1,150	22.22	
Poor	3117	24.15	805	25.83		3293	29.1	882	26.78	
Missing	701	5.43	170	24.25		495	4.37	104	21.01	

Overall, the coverage of population without any residential toilets decreased greatly, with coverage at 29.45% (3899/13240) in 2011, 26.19% (3629/13856) in 2013, 21.63% (2792/12909) in 2015, and 23.58% (2668/11316) in 2018 among the middle-aged and elderly population in rural China. The average age of those without residential toilets was 59.91, 59.69, 62.19, and 64.52 in 2011, 2013, 2015, and 2018, respectively. Chi square tests revealed that age, education level, marital status, household size, annual per capita household consumption, region, having running water, self-reported health, ADLs, and region had significant associations with toilet ownership. From 2011 to 2018, the coverage of residential toilets among those aged ≥ 45 years in the rural Northeast was much lower than that in other rural regions (Table 1 and 2).

Among the samples of people with residential toilets, the coverage of flushable toilets increased steadily from 2011 to 2018, with coverage at 39.15% (3650/9323) in 2011, 45.06% (4595/10198) in 2013, 43.50% (3431/7888) in 2015, and 50.23% (3067/6106) in 2018. The coverage of toilets with seats also witnessed a similar trend, starting from 14.50% (1354/9341) in 2011, to 17.94% (1835/10227) in 2013, to 21.90% (2216/10117) in 2015, and to 29.37% (2540/8648) in 2018.

Table 3 and 4 show the results of logistic regression for access to toilets, flushable toilets, and toilets with seats in the residence. The multicollinearity test found that the Variance Inflation Factor in all logit models was below 1.2. That means our logit models did not have multicollinearity. We found that being female, higher level of education, having residential running water, higher annual per capita household consumption, larger household size, and better health status were significantly associated with an increased likelihood of residential toilet ownership. Compared with those from the East, respondents from the Northeast were less likely to own residential toilets. And compared with those from the East, those from the West were more likely to own toilets in 2011, but were less likely to have toilets in 2018. In addition, we revealed that having residential running water, higher annual per capita household consumption, and larger household size were positively significantly associated with the likelihood of access to flushable toilets and toilets with seats. Compared with those from the Eastern region, those from other rural regions of China were less likely to have toilets with seats, and those from Northeastern and Central China were less likely to possess flushable toilets. Compared with those with good health, those with fair and poor health were less likely to own toilets with seats. We also found that those with ADL disability were less likely to own flushable toilets and the married were less likely to own toilets with seats.

**Table 3:** Multivariable logistic regression of factors associated with access to toilet in the residence

	Odd ratio(95% confidence interval)			
	2011 (*n* = 13,240)	2013 (*n* = 13,856)	2015 (*n* = 12,909)	2018 (*n* = 11,316)
**Gender**				
Male	1	1	1	1
Female	1.142^**^ (1.048 to 1.246)	1.056(0.967 to 1.153)	1.019(0.922 to 1.125)	1.151^**^ (1.040 to 1.275)
**Age**				
45–59	1	1	1	1
60–69	0.969(0.878 to 1.070)	0.994(0.902 to 1.097)	0.991(0.887 to 1.107)	0.960(0.857 to 1.076)
70 and above	1.041(0.920 to 1.179)	0.984(0.873 to 1.110)	0.881(0.772 to 1.006)	0.991(0.866 to 1.134)
**Education Level**				
Illiterate	0.800^*^ (0.661 to 0.967)	0.847(0.694 to 1.035)	0.989(0.787 to 1.243)	0.759^*^(0.602 to 0.958)
Primary or middle school	1.042(0.874 to 1.241)	0.939(0.781 to 1.129)	0.979(0.794 to 1.207)	0.946(0.764 to 1.170)
High school or above	1	1	1	1
**Marital status**				
Married	1	1	1	1
Divorced/widowed/never married	0.973(0.843 to 1.123)	1.026(0.881 to 1.194)	0.925(0.787 to 1.086)	1.140(0.979 to 1.327)
**Region**				
Northeastern	0.152^***^(0.125 to 0.185)	0.210^***^(0.176 to 0.252)	0.151^***^(0.125 to 0.183)	0.148^***^(0.121 to 0.180)
Western	1.432^***^(1.292 to 1.586)	0.905(0.815 to 1.005)	0.937(0.833 to 1.053)	0.679^***^(0.604 to 0.764)
Central	1.506^***^ (1.353 to 1.676)	1.073(0.959 to 1.201)	1.189^**^(1.048 to 1.351)	1.034(0.910 to 1.175)
Eastern	1	1	1	1
**Having running water**				
No	1	1	1	1
Yes	2.693^c^(2.474 to 2.931)	1.878^c^(1.721to 2.050)	1.776^c^(1.605 to 1.964)	2.322^c^(2.096 to 2.572)
**Household size**				
Living alone	1	1	1	1
2–4 people	1.439^**^ (1.170 to 1.771)	1.669^***^(1.338 to 2.081)	1.671^***^(1.338 to 2.088)	1.697^***^ (1.341 to 2.149)
5 people and above	1.786^***^(1.443 to 2.211)	2.516^***^(1.999 to 3.165)	2.494^***^ (1.920 to 3.241)	2.177^***^ (1.663 to 2.849)
**SES**				
Lowest 25%	1	1	1	1
Lower 25%	1.088(0.975 to 1.214)	1.073(0.954 to 1.206)	1.115(0.979 to 1.269)	1.242^**^(1.092 to 1.411)
Higher 25%	1.133^*^(1.013 to 1.267)	1.132^*^(1.005 to 1.275)	1.276^***^ (1.117 to 1.458)	1.411^***^(1.236 to 1.611)
Highest 25%	1.454^***^(1.293 to 1.634)	1.226^**^(1.084 to 1.386)	1.405^***^(1.226 to 1.611)	1.646^***^ (1.434 to 1.889)
**ADL**				
No ADL disability	1	1	1	1
With ADL disability	0.987(0.833 to 1.169)	0.918(0.767 to 1.099)	0.985(0.817 to 1.187)	0.859(0.721 to 1.024)
**Self-reported health**				
Good	1	1	1	1
Fair	1.047(0.943 to 1.162)	1.044(0.938 to 1.162)	1.003(0.889 to 1.132)	1.095(0.968 to 1.239)
Poor	0.804^***^ (0.717 to 0.901)	0.812^**^ (0.720 to 0.915)	0.754^***^(0.658 to 0.863)	0.878(0.768 to 1.004)
**Constant**	0.681^*^(0.467 to 0.994)	1.087(0.722 to 1.635)	1.652^*^(1.064 to 2.564)	0.819(0.527 to 1.273)

^*^P < 0.05

^**^P < 0.01

^***^P < 0.001

**Table 4:** Multivariable logistic regression of factors associated with access to flushable toilets and toilets with seats among those with toilets in the residence in 2018

	Odd ratio(95% confidence interval)	
	Outcome variable: flushable toilets(*n* = 6106)	Outcome variable: toilets with seats (*n* = 8648)
**Gender**		
Male	1	1
Female	1.031(0.919 to 1.158)	1.026(0.918 to 1.147)
**Age**		
45–59	1	1
60–69	0.949(0.836 to 1.078)	0.901(0.797 to 1.017)
70 and above	0.930(0.797 to 1.084)	1.066(0.919 to 1.237)
**Education Level**		
Illiterate	0.877(0.677 to 1.136)	1.005(0.797 to 1.268)
Primary or middle school	1.027(0.813 to 1.297)	0.854(0.696 to 1.049)
High school or above	1	1
**Marital status**		
Married	1	1
Divorced/widowed/never married	1.174(0.990 to 1.393)	1.183*(1.004 to 1.394)
**Region**		
Northeastern	0.209***(0.134 to 0.327)	0.694*(0.504 to 0.956)
Western	0.960(0.838 to 1.100)	0.262***(0.231 to 0.298)
Central	0.723***(0.629 to 0.830)	0.333***(0.294 to 0.378)
Eastern	1	1
**Having running water**		
No	1	1
Yes	1.643***(1.445 to 1.867)	3.065***(2.587 to 3.632)
**Household size**		
Living alone	1	1
2–4 people	1.678**(1.244 to 2.263)	1.616**(1.200 to 2.176)
5 people and above	2.598***(1.867 to 3.615)	1.795***(1.295 to 2.489)
**SES**		
Lowest 25%	1	1
Lower 25%	1.320***(1.138 to 1.530)	1.469***(1.252 to 1.724)
Higher 25%	1.457*** (1.254 to 1.693)	1.600*** (1.365 to 1.874)
Highest 25%	2.196***(1.869 to 2.580)	2.926***(2.505 to 3.419)
**ADL**		
No ADL disability	1	1
With ADL disability	0.623*** (0.499 to 0.778)	1.103(0.889 to 1.369)
**Self-reported health**		
Good	1	1
Fair	1.150(0.998 to 1.325)	0.855*(0.752 to 0.970)
Poor	0.872(0.746 to 1.019)	0.703***(0.606 to 0.815)
**Constant**	0.278*** (0.165 to 0.466)	0.124*** (0.075 to 0.205)

*P<0.05; **P<0.01; ***P<0.001

## Discussion

This study made an important contribution to the existing research, as it is one of the few studies focusing on trends of and factors associated with residential toilet access in rural China in recent years. Our results showed that from 2011 to 2018 the proportion of people aged ≥ 45 years in rural China without residential toilet access dropped by about 6%. This result is similar to previous studies showing that in rural areas both the coverage of sanitary toilets and the equity in sanitation rose from 2008 to 2013 [26, 27, 29]. And our result is also in line with both the estimates of sanitary toilet coverage in rural China from National Health Commission of the People’s Republic of China (81.8%) [5] and coverage figures of at least basic sanitation services in rural China from the World Health Organization (76%) in 2017. The progress in sanitation improvement in rural China is largely due to the series of initiatives taken by the government. Since 2009, the government has increased remarkably its central investment to sanitation improvement every year [27]. However, there is still a gap between the current coverage of toilets in rural areas identified in this study and the goal set in the National Environmental Sanitation Action Plan (2015–2020), i.e. the coverage of sanitary toilets would reach 85% by 2020 and 100% by 2030. Considering the gap between the current levels of toilet ownership and the goals, special efforts are still needed to improve sanitation in rural China further.

We also found that the coverage of flushable toilets and toilets with seats rose substantially from 2011 to 2018 among the middle-aged and elderly population in rural China, which indicates that the quality of residential toilets in rural China have improved significantly. Making toilets flushable is not one of the fundamental goals of the toilet revolution in China [4], since some parts of rural China lack water. However, flushable toilets are valuable and convenient for the elderly. So are toilets with seats. Therefore, more and more middle-aged and elderly people choose to use flushable toilets and toilets with seats in rural China.

This study further identified the factors associated with access to toilets, flushable toilets, and toilets with seats in the residence. We found that in 2018 the odds for owners of residential toilets without education were 21.3% lower than the odds for owners of residential toilets with a high school or higher education. This is consistent with findings in one province in rural China, Vietnam, and East Africa showing that less-educated families were less likely to access toilets [22, 24, 28]. Meanwhile, we found that the odds for females owning residential toilets were about 11% higher than the odds for males in 2011 and 2018. Studies have shown that in rural settings having one’s own toilet results in greater protection of women’s privacy [33]. Therefore, women have a high willingness to construct residential toilets. In line with quantitative studies in rural Indonesia showing that having access to water throughout the year significantly influenced toilet ownership [23], this study proved that having running water was positively associated with owning toilets, flushable toilets, and toilets with seats in the residence. We suggest that the government should strengthen water improvement together with toilet renovation in order to comprehensively enhance the health of rural residents.

In addition, we revealed that compared with residents from the rural East, residents from the Northeast were less likely to have residential toilets from 2011 to 2018. And compared with residents from the rural East, residents from the rural West were more likely to own toilets in 2011, but were less likely to have toilets in 2018. The coverage ranking of residential toilets among the four regions from 2011 to 2018 is similar to the coverage ranking of harmless residential toilets reported in China Environmental Statistics Yearbook. In 2011 the coverage of harmless sanitary toilets in rural Eastern, Central, Western, and Northeastern China stood at 35.35%, 42.27%, 41.36%, and 17.93%, respectively [34]. In 2017 the coverage of harmless sanitary toilets in rural Eastern, Central, Western, and Northeastern China stood at 83.04%, 52.99%, 56.00%, and 30.69%, respectively [35]. The high coverage of residential toilets among the middle-aged and elderly and the high coverage of harmless sanitary toilets among the general population in 2011 in rural Western and Central China might be because during the 3-year health reform program from 2009 to 2011 central investments for sanitation improvement had been concentrated more on these regions [27]. When comparing the data in this study with the data from China Environmental Statistics Yearbook, one needs to bear in mind that our study focused on residential toilets among the population aged ≥ 45 years, whereas China Environmental Statistics Yearbook focused on harmless sanitary toilets among the general population. This study also found that compared with residents from the rural East, residents from other rural regions of China were less likely to have toilets with seats, and those from Northeastern and Central China were less likely to possess flushable toilets. In general, the economic development level of eastern China is higher than that of other regions of China. Residents from the rural East on average have more resources to invest in constructing flushable toilets and toilets with seats, which cost more than general toilets. Further in-depth analysis of regional differences in toilet improvement in China is still needed.

Interestingly, we demonstrated that the smaller the household size, the lower the probability of possessing toilets, flushable toilets, and toilets with seats in the residence. This is in agreement with findings from East Africa showing that smaller families were prone to have no toilets [22]. Moreover, in line with previous studies [21, 24, 25, 28], our study identified that those members of the population aged ≥ 45 years with higher annual per capita household consumption were more likely to own toilets, flushable toilets, and toilets with seats in the residence in rural China. This could be explained by the fact that families with higher household socioeconomic status could afford the economic costs of toilet construction and renovation, so they are more likely to invest in sanitation improvement.

In addition, this study revealed that those in the population aged ≥ 45 years with a poorer health status were associated with a decreased likelihood of owning toilets and toilets with seats in the residence in rural China. This finding is consistent with that of a previous study finding that adults with better health status were more likely to live in a clean environment with good sanitation facilities [36]. We also uncovered that the odds for people with ADL disability having flushable toilets were 37.7% lower than the odds for those without ADL disability. The above results are worrisome. Deprivation of toilets, flushable toilets, and toilets with seats in the residence may make the elderly with poorer health status and ADL disability face more health risks, and possibly further increasing their vulnerabilities and health deterioration.

### Limitations

A few limitations needed to be acknowledged in this study. First, the total numbers of people answering the questions on flushable toilets and toilets with seats were not the same. Therefore, the denominators of the coverage of flushable toilets and toilets with seats were different. Second, CHARLS contained limited information on toilets. Besides indicators of flushable toilets and toilets with seats, we were unable to analyze other indicators related to elderly-oriented toilets in rural China, such as the coverage of toilet handles. CHARLS also lacked data on attitudes towards toilets or intention to build toilets. Therefore, we could not analyze the relationship between respondents’ perceptions of toilets and actual toilet ownership. Future targeted studies are needed in this regard. Third, similar with other high-impact studies on factors influencing hypertension and diabetes [37], as well as access to sanitation facilities [28], etc., we did logistic regression between residential toilet ownership and the selected explanatory variables in each year, since the aim of this study was to explore the association between them but not to set up a causal relationship. Future studies are needed to utilize the longitudinal data analysis methods to explore the related topics based in China.

## Conclusions

This study found that the coverage of residential toilets in rural China among people aged ≥ 45 increased from 2011 to 2018. A similar trend was observed for the coverage of both flushable toilets and toilets with seats. We also identified the subgroups who were more likely to be deprived of residential toilets, defined by being male, lower levels of education, lower annual per capita household consumption, without running water in the residence, smaller household size, and poorer health status. We suggest to the government that in order to ensure universal coverage of sanitation facilities for the whole of the Chinese population, these subgroups should be the targeted population when designing further toilet improvement interventions in rural China.

## Supplementary Information

**Additional file 1.** The sample in each selected province in each year

## Data Availability

We used the CHARLS data from 2011 to 2018, which are available upon application. Link: http://charls.pku.edu.cn/index/en.html.

## Abbreviations

CHARLS: China Health and Retirement Longitudinal Study
SDGs: Sustainable Development Goals
LMICs: Low- and middle-income countries
ADLs: Activities of daily living
CNY: Chinese Yuan
