# Supplementary material for: Trends of and factors associated with access to residential toilets among the middle-aged and elderly in rural China from 2011 to 2018
Source: BMC Public Health. 2022 Mar 2;22:421. doi: 10.1186/s12889-022-12739-3 (PMC8889666; doi:10.1186/s12889-022-12739-3)
Supplement: Supplementary file 1 — Additional file 1. The sample in each selected province in each year [file 12889_2022_12739_MOESM1_ESM.docx]

Appendix Table 1 The sample in each selected province in each year

|  | 2011 | | 2013 | | 2015 | | 2018 | |
| --- | --- | --- | --- | --- | --- | --- | --- | --- |
|  | n | % | n | % | n | % | n | % |
| Yunnan | 883 | 6.67 | 902 | 6.51 | 835 | 6.47 | 734 | 6.49 |
| Fujian | 482 | 3.64 | 483 | 3.49 | 446 | 3.45 | 355 | 3.14 |
| Qinghai | 148 | 1.12 | 152 | 1.10 | 142 | 1.10 | 133 | 1.18 |
| Sichuan | 1,273 | 9.61 | 1,370 | 9.89 | 1,297 | 10.05 | 1,111 | 9.82 |
| Hebei | 572 | 4.32 | 622 | 4.49 | 568 | 4.40 | 530 | 4.68 |
| Jiangxi | 529 | 4.00 | 564 | 4.07 | 535 | 4.14 | 470 | 4.15 |
| Xinjiang | 33 | 0.25 | 37 | 0.27 | 35 | 0.27 | 24 | 0.21 |
| Beijing ^1^ | 4 | 0.03 | 4 | 0.03 | 2 | 0.02 | 0 | 0.00 |
| Inner Mongolia | 567 | 4.28 | 595 | 4.29 | 529 | 4.10 | 455 | 4.02 |
| Jiangsu | 663 | 5.01 | 656 | 4.73 | 626 | 4.85 | 539 | 4.76 |
| Chongqing ^1^ | 167 | 1.26 | 172 | 1.24 | 162 | 1.25 | 140 | 1.24 |
| Gansu | 363 | 2.74 | 394 | 2.84 | 365 | 2.83 | 325 | 2.87 |
| Heilongjiang | 96 | 0.73 | 129 | 0.93 | 117 | 0.91 | 102 | 0.90 |
| Guangdong | 818 | 6.18 | 795 | 5.74 | 743 | 5.76 | 615 | 5.43 |
| Liaoning | 343 | 2.59 | 376 | 2.71 | 342 | 2.65 | 300 | 2.65 |
| Shanxi | 454 | 3.43 | 466 | 3.36 | 431 | 3.34 | 388 | 3.43 |
| Shanghai ^1^ | 8 | 0.06 | 10 | 0.07 | 12 | 0.09 | 8 | 0.07 |
| Tianjin ^1^ | 118 | 0.89 | 99 | 0.71 | 81 | 0.63 | 69 | 0.61 |
| Zhejiang | 531 | 4.01 | 581 | 4.19 | 562 | 4.35 | 498 | 4.40 |
| Jilin | 220 | 1.66 | 225 | 1.62 | 207 | 1.60 | 188 | 1.66 |
| Guangxi | 435 | 3.29 | 450 | 3.25 | 393 | 3.04 | 351 | 3.10 |
| Anhui | 735 | 5.55 | 713 | 5.15 | 697 | 5.40 | 616 | 5.44 |
| Hubei | 433 | 3.27 | 470 | 3.39 | 407 | 3.15 | 344 | 3.04 |
| Shaanxi | 411 | 3.10 | 419 | 3.02 | 398 | 3.08 | 361 | 3.19 |
| Shandong | 1,114 | 8.41 | 1,247 | 9.00 | 1,184 | 9.17 | 1,066 | 9.42 |
| Henan | 1,096 | 8.28 | 1,165 | 8.41 | 1,071 | 8.30 | 962 | 8.50 |
| Hunan | 566 | 4.27 | 582 | 4.20 | 556 | 4.31 | 487 | 4.30 |
| Guizhou | 178 | 1.34 | 178 | 1.28 | 166 | 1.29 | 145 | 1.28 |
| Total | 13240 | 100% | 13856 | 100% | 12909 | 100 | 11,316 | 100% |

^1^ Beijing, Chongqing, Shanghai, and Tianjin are municipalities, which are provincial administrative regions in China.
